# Interpretive machine learning predicts short-term mortality risk in elderly sepsis patients

**DOI:** 10.3389/fphys.2025.1549138

**Published:** 2025-03-26

**Authors:** Xing-Yu Zhu, Zhi-Meng Jiang, Xiao‐ Li, Zi-Wen Lv, Jian-Wei Tian, Fei-Fei Su

**Affiliations:** ^1^ Graduate School of Hebei North University, Zhangjiakou, Hebei, China; ^2^ Department of Cardiovascular Medicine, Chinese People’s Liberation Army Air Force Medical Center, Beijing, China

**Keywords:** sepsis, machine learning, shapley additive explanations, local interpretable model-agnostic explanations, XGBoost

## Abstract

**Backgrounds:**

Sepsis is a leading cause of in-hospital mortality. However, its prevalence is increasing among the elderly population. Therefore, early identification and prediction of the risk of death in elderly patients with sepsis is crucial. The objective of this study was to create a machine learning model that can predict short-term mortality risk in elderly patients with severe sepsis in a clear and concise manner.

**Methods:**

Data was collected from the MIMIC-IV (2.2). It was randomly divided into a training set and a validation set using a 7:3 ratio. Mortality predictors were determined through Recursive Feature Elimination (RFE). A prediction model for 28 days of ICU stay was built using six machine-learning algorithms. To create a comprehensive and nuanced model resolution, Shapley Additive Explanations (SHAP) and Local Interpretable Model-Agnostic Explanations (LIME) were used to systematically interpret the models at both a global and detailed level.

**Results:**

The study involved the analysis of 4,056 elderly patients with sepsis. A feature recursive elimination algorithm was utilized to select eight variables out of 49 for model development. Six machine learning models were assessed, and the Extreme Gradient Boosting (XGBoost) model was found to perform the best. The validation set achieved an AUC of 0.88 (95% CI: 0.86–0.90) and an accuracy of 0.84 (95% CI: 0.81–0.86) for this model. To examine the roles of the eight key variables in the model, SHAP analysis was employed. The global ranking order was made evident, and through the use of LIME analysis, the weights of each feature range in the prediction model were determined.

**Conclusion:**

The study’s machine learning prediction model is a dependable tool for forecasting the prognosis of elderly patients with severe sepsis.

## Introduction

Sepsis has the potential to lead to multiple organ dysfunction syndrome (MODS) and in severe cases, even death. It is a major factor contributing to mortality and morbidity worldwide (Rudd et al., 2020; Singer et al., 2016). In the late 1970s, it was estimated that approximately 164,000 new cases of sepsis occurred annually in the United States (Martin et al., 2003). It is worth noting that the incidence of sepsis has been increasing globally, not just in the United States (Walkey et al., 2013; Fleischmann et al., 2016; Rhee et al., 2017). Estimates suggest that sepsis mortality rates can vary between 10 and 52 percent (Martin et al., 2003; Kaukonen et al., 2014; Li A. et al., 2022; Epstein et al., 2016). It has been observed that the age group of 65 years and over is more vulnerable to sepsis, with a significant percentage of cases, occurring in this demographic. Considering the ongoing demographic shift towards an aging population, it is anticipated that the incidence of sepsis will rise in the future (Martin et al., 2003; Kaukonen et al., 2014; Angus et al., 2001; Giacomello and Toniolo, 2021). Identifying and predicting mortality risk in elderly septicaemic patients at an early stage is of utmost importance. This information can provide a valuable reference for clinicians to improve patient survival and prognosis through timely and effective therapeutic measures. Although some machine learning-based predictive models have been developed, their limited interpretability has hindered their application in clinical practice.

Rapid advancements in machine learning (ML) have provided powerful tools for extracting complex patterns, assisting clinical decision-making, and improving patient prognosis prediction in medicine. This section briefly outlines several prevalent ML models and their applications in medical prognosis. Logistic regression, a classical statistical learning method, is suitable for binary classification problems. Its model is concise and efficient, offering high interpretability and widespread application in areas such as cardiovascular disease risk assessment (Zabor et al., 2022); however, it struggles with complex non-linear relationships. Support vector machines (SVM), employing kernel methods to map data into high-dimensional space, excel at handling non-linear classifications (Zhou, 2022). While demonstrating superior performance in predicting breast cancer patient survival, they entail complex hyperparameter tuning and high computational costs (Sarkar and Mali, 2023). Neural networks, leveraging multiple layers of neurons to simulate non-linear mappings, are widely used in EHR data analysis and medical image processing (Manimegalai et al., 2022). Nevertheless, their “black box” nature and reliance on large annotated datasets remain challenges. Multilayer perceptrons (MLP), a foundational neural network architecture, exhibit strong performance in image segmentation due to their flexibility; however, their efficiency diminishes when confronted with multimodal data (Gao et al., 2023; Zhang et al., 2023). Naive Bayes (NB), based on Bayes’ theorem and assuming feature independence, boasts high computational efficiency. Demonstrating excellent performance in genomic data analysis and cancer patient stratification prediction, its performance is nevertheless affected by feature correlations (Das and Dutta, 2020; Lu et al., 2024). Extreme Gradient Boosting (XGBoost), employing ensemble learning to construct robust predictive models, shows superior performance in predicting mortality risk in heart failure patients (Li J. et al., 2022). While its efficient feature handling capabilities are beneficial for imbalanced datasets, the risk of overfitting in small datasets necessitates careful consideration (Takefuji, 2025). These models, each with its unique strengths, provide valuable support for data analysis and decision-making in medical prognosis. Future advancements, focusing on enhancing model explainability and effectively integrating multimodal data, promise to further unlock their clinical potential.

Research in critical care medicine has been exploring prognostic modeling for sepsis patients. It has been found that commonly used serological indices, such as calcitonin, platelet, and lactate levels, may have limited prognostic value in assessing the effect of sepsis (Brunkhorst et al., 2000; Gattinoni et al., 2019). Sepsis is a clinical syndrome that presents a wide range of biological characteristics. As a result, it can be challenging to fully reflect the patient’s condition based on individual indicators alone (Hernandez et al., 2019). The efficacy of emerging machine learning techniques in comparison to traditional means of prediction is contingent on the nature of the dataset and the field in which they are employed. It has been hypothesized that machine learning techniques may be more appropriate for highly innovative fields with large amounts of data (Rajula et al., 2020). By meticulously designing and optimizing algorithms, they are capable of learning from large and intricate datasets to uncover more profound associations between patients’ clinical indicators and prognostic outcomes. Nevertheless, it is important to acknowledge that machine learning models in clinical practice may have limitations stemming from the lack of clarity and intuition in the decision-making process (Karim et al., 2023).

When constructing algorithmic models, it is common to use precision and recall of the test set as benchmarks for measuring model strength. However, when communicating with non-professionals, relying on a single value may not be practical. Therefore, it is important to demonstrate the internal logic and principles of the model to enhance its credibility. It is worth noting that certain models are not always constructed based on clear rules. Some black-box models, despite their higher predictive accuracy, may be opaque and unable to provide a specific decision-making basis. This can make it challenging for the general public to comprehend and have faith in their predictions. Thankfully, there are now tools available such as SHAP and lime that can provide valuable insights into the decision-making processes of machine learning models (Hilton et al., 2020; Lundberg et al., 2020). These tools can facilitate a deeper comprehension of how machine learning models operate, which could lead to their expanded implementation in clinical practice.

This investigation aims to develop a machine learning model that can predict the short-term risk of death in elderly patients with sepsis with accuracy. Advanced SHAP and lime technical tools will be utilized to comprehensively analyze and explore the model at both the holistic and local levels.

## Methods

### Database

This retrospective study utilized data from the publicly available electronic health record dataset MIMIC-IV(2.2) (Johnson et al., 2023). The database was created by the Computational Physiology Laboratory of the Massachusetts Institute of Technology (MIT), the Beth Israel Deaconess Medical Centre (BIDMC) at Harvard Medical School, United States, and Philips Healthcare. Patient information was collected and research resources were created with approval from the Beth Israel Deaconess Medical Centre’s Institutional Review Board, which waived the need for informed consent and supported the data-sharing initiative (Johnson et al., 2023; Goldberger et al., 2000). The study adheres rigorously to the tenets set forth in the Declaration of Helsinki. Zi-wen Lv, the author of this study, has completed the CITI course and passed the ‘Conflict of Interest’ and ‘Data or Specimen Research Only’ examinations (ID: 55,109,354). As a result, we are authorized to use this database.

### Study population

This study leverages the advantages of the heterogeneous and multisource clinical data contained in the MIMIC-IV (version 2.2) database, which provides comprehensive electronic health records (EHRs) and longitudinal follow-up information to support the refined analytical requirements of prognostic modeling. Using Structured Query Language (SQL) and the Navicat 16.3.8 software platform, a systematic screening and extraction of raw datasets meeting the inclusion criteria was conducted to construct an association framework between high-dimensional features and clinical outcomes. During data processing, patients with septicemia in the MIMIC database were accurately identified and classified using specific codes, such as R6520, R6521, and 99,592. Data on patients, including socio-demographic characteristics, vital signs, laboratory parameters, and complications, was extracted using structured query language (Yang et al., 2020). To determine the study sample, strict inclusion and exclusion criteria were applied. Inclusion criteria required patients to be admitted to the ICU with a confirmed diagnosis of sepsis and to be 65 years old or older. Exclusion criteria were applied to patients who were not first-time ICU admissions and those with more than 30% of missing data variables.

### Approaching the issue of missing data

Data loss is a known issue in clinical trials, which may impact the integrity of the original data set and potentially weaken the robustness and validity of study findings (Sterne et al., 2009). Multiple imputation is a commonly used method for dealing with missing data in interpolation. This method provides valid estimates while also accounting for the uncertainty associated with missing data (Pedersen et al., 2017; Heymans and Twisk, 2022). To address the issue of missing data, a multiple imputation approach was employed. Variables such as triglycerides and total cholesterol, which had a missing rate of over 30%, were excluded from the analysis. The remaining variables were refined using multiple imputation techniques. The data-filling task was completed using the ‘mice’ package in R4.2.3. Through engagement with clinical experts specializing in sepsis management, we systematically assessed the relevance and potential impact of excluded variables. Their expert consensus confirmed that the removal of these variables would not compromise the prognostic accuracy or introduce significant bias in our analysis of sepsis outcomes.

### Statistical analyses

The data were analyzed using both R languages (version 4.2.3). For continuous variables that follow a normal distribution, the mean and standard deviation (*x̅*±*s*) were used to describe their concentration and dispersion trends. For continuous variables that do not follow a normal distribution, the median and interquartile range (M(Q1, Q3)) were used to characterize their distributions. Differences between groups for normally distributed variables were compared using the t-test, while the Mann-Whitney U test was used for non-normally distributed variables. The distributions of categorical variables were visualized as percentages. The chi-square test was used to determine significant associations between categories. Additionally, we analyzed the non-linear relationship between characteristic variables and the risk of short-term mortality in elderly patients with sepsis using restricted cubic bars.

### Development and validation of predictive models

While models with rich features often outperform those with fewer features in terms of accuracy, this is not always the practice case. In clinical applications, it is important to carefully consider the number of features used in a model, as simply increasing the number of features does not always lead to improved performance. This is because irrelevant or redundant features may negatively impact the model’s accuracy, and an excessive number of features may result in overfitting. The study employed a Recursive Feature Elimination (RFE) algorithm to develop a model based on 49 variables that were strongly associated with short-term mortality outcomes.

The RFE algorithm is a model-based strategy for selecting features. It screens the best feature combinations by iteratively training the model and gradually eliminating the features with the lowest weights. To ensure a more accurate measurement of performance fluctuation during the feature selection process, this study incorporates a layer of resampling (10-fold cross-validation) outside the RFE algorithm. The algorithm identified eight features that had a significant impact on predicting short-term risk of mortality in elderly patients with severe sepsis. This study utilized six machine learning models, specifically Logistic Regression, Neural Network, Support Vector Machine, Multilayer Perceptron, Naive Bayes, and Extreme Gradient Boosting, to create effective prediction models. The patient dataset was randomly sampled and split into training and validation sets in a 7:3 ratio during the development of the prediction model. To ensure accurate parameter tuning and model resilience, a tenfold cross-validation technique was employed. The models’ predictive performance was assessed using the area under the working characteristic curve of the subjects as the primary index. Furthermore, a detailed evaluation of each model was conducted, including sensitivity, specificity, positive predictive value, negative predictive value, recall, precision, and F1 score. To provide a more comprehensive representation of the optimal models’ performance, we have included standard curves and decision analysis curves (DCA) (Van Calster et al., 2018).

### Tools for interpreting machine learning

This study employs two model interpretation methods, namely, SHAP and lime, to conduct a thorough analysis of the risk prediction model that we have developed. We extensively investigate the contribution of each clinical variable in the model. It is worth noting that SHAP is a commonly used tool for evaluating the impact of features in machine learning models (Li J. et al., 2022; Fahmy et al., 2022). The main concept is to measure the impact of each feature on the model’s final output, providing a comprehensive understanding of the ‘black box model’ from both a global and local perspective (Lundberg and Lee, 2017). The SHAP value provides an accurate representation of the positive and negative impact that each predictor has on the target variable (Lundberg and Lee, 2017). Lime is an advanced algorithm that can generate interpretable models for any classifier or regressor, enabling accurate interpretation of predicted results through local approximation. This is accomplished by training a local substitution model to provide a detailed interpretation of a single instance (Peng and Menzies, 2021). Lime can provide a comprehensive understanding of the impact of various characteristic variables on the predictive model. This can help to ensure that the model is accurate and reliable.

## Result

### Patient characteristics

**FIGURE 1:**
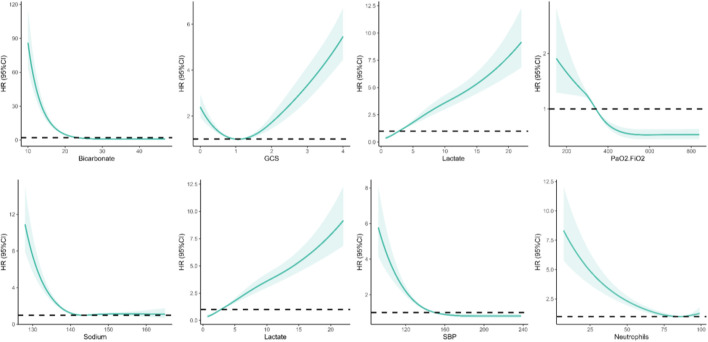
Non-linear relationships among eight screened variables and the risk of death from sepsis in elderly patients.

The study involved a total of 4,056 elderly patients diagnosed with sepsis, out of which 1,259 patients passed away within 28 days. These patients were randomly assigned to either the training set (2,865 patients) or the validation set (1,191 patients) in a 7:3 ratio. Table 1 presents a detailed comparison of the baseline characteristics between the group that passed away within 28 days and the non-death group in the training set. During the study, we employed a recursive feature elimination algorithm to determine the eight most closely related variables to short-term mortality risk. These variables, listed in order of importance, are Bicarbonate, GCS, Lactate, Platelets, PaO2/FiO2, SBP, Sodium, and upon analyzing Table 1, it becomes evident that these eight core indicators are statistically significant when comparing the fatal and non-fatal groups in the training set. The study examined the correlation between eight core indicators and the risk of death in elderly patients with sepsis, utilizing restricted cubic spline analysis. The findings suggest a significant non-linear correlation between these metrics and the risk of death in elderly patients with sepsis (p-non-linear <0.001). For further details, please refer to Figure 1.

**TABLE 1:** Baseline characteristics of the training set.

Variables	Total	Survival within 28 days	Death within 28 days	*p*
Number (sample size)	2865	1975	890	
Sex, n (%)				0.204
Female	1316 (46)	891 (45)	425 (48)	
Male	1549 (54)	1084 (55)	465 (52)	
Ethnicity, n (%)				<0.001
Black	259 (9)	183 (9)	76 (9)	
White	2041 (71)	1445 (73)	596 (67)	
Others	565 (20)	347 (18)	218 (24)	
Age, Median (Q1, Q3)	77 (71, 85)	77 (71, 84)	78 (72, 85)	0.006
BMI (kg/m2), Median (Q1,Q3)	27.02 (24.16, 31.25)	27.16 (24.35, 31.57)	26.7 (23.68, 30.75)	0.003
Weight (kg), Median (Q1, Q3)	75.5 (63, 90.6)	76.1 (63.5, 91)	73.75 (62.12, 89.57)	0.019
Height (cm), Median (Q1, Q3)	168 (162.69, 173.18)	168.07 (163, 173.61)	166.75 (160.57, 173)	<0.001
SBP (mmHg), Median (Q1,Q3)	150 (135, 167)	153 (138, 169)	143 (130, 160)	<0.001
PaO2/FiO2, Median (Q1,Q3)	340.84 (283.33, 419.05)	359.15 (293.33, 422.79)	293.33 (283.33, 379.46)	<0.001
GCS, Median (Q1, Q3)	1 (1, 3)	1 (1, 2)	2 (1, 4)	<0.001
Heart failure, n (%)	1255 (44)	857 (43)	398 (45)	0.534
Respiratory failure, n (%)	1419 (50)	847 (43)	572 (64)	<0.001
Hypertension, n (%)	1110 (39)	808 (41)	302 (34)	<0.001
AMI, n (%)	431 (15)	288 (15)	143 (16)	0.331
Atrial fibrillation, n (%)	1341 (47)	910 (46)	431 (48)	0.26
AKI, n (%)	1976 (69)	1308 (66)	668 (75)	<0.001
Diabetes, n (%)	1188 (41)	816 (41)	372 (42)	0.841
RBC (m/uL), Median (Q1,Q3)	3.71 (3.3, 4.15)	3.75 (3.33, 4.16)	3.62 (3.23, 4.11)	<0.001
Lactate (mmol/L), Median (Q1,Q3)	2.8 (1.9, 4.8)	2.4 (1.7, 3.7)	4.4 (2.6, 8.5)	<0.001
WBC (K/uL), Median (Q1,Q3)	18.8 (13.4, 26.1)	18.1 (13.15, 25.1)	20.2 (14.62, 29)	<0.001
Sodium (mEq/L), Median (Q1,Q3)	144 (141, 148)	144 (141, 148)	143 (139, 148)	<0.001
Platelets (K/uL), Median (Q1,Q3)	273 (186, 386)	296 (205, 415)	230 (144, 318)	<0.001
Total_Calcium (mg/dL), Median (Q1, Q3)	8.8 (8.4, 9.3)	8.9 (8.4, 9.3)	8.8 (8.2, 9.4)	<0.001
Hemoglobin (g/L), Median (Q1,Q3)	11 (9.8, 12.3)	11.1 (9.8, 12.3)	10.8 (9.6, 12.2)	0.01
RDW (%), Median (Q1,Q3)	16.8 (15.1, 19.1)	16.6 (15, 18.8)	17.4 (15.62, 19.7)	<0.001
ALT (IU/L, Median (Q1,Q3)	49 (22, 136)	46 (22, 117.5)	56 (23, 252.75)	<0.001
Lymphocytes (%), Median (Q1,Q3)	11.1 (7, 16.6)	11.7 (7.5, 17)	10 (6, 15.2)	<0.001
ALP (IU/L), Median (Q1,Q3)	131 (85, 215)	130 (84, 213.5)	135.73 (86, 220.75)	0.188
Potassium (mEq/L), Median (Q1,Q3)	4.9 (4.4, 5.5)	4.8 (84, 213.5)	5.1 (4.6, 5.8)	<0.001
Neutrophils (%), Median (Q1,Q3)	86 (80.4, 90.5)	86 (81, 90.5)	86.17 (79, 90.5)	0.054
INR, Median (Q1,Q3)	1.7 (1.4, 2.7)	1.6 (1.3, 2.4)	2 (1.5, 3.1)	<0.001
HCT (%), Median (Q1,Q3)	34.2 (30.8, 38.1)	34.3 (30.9, 38)	33.9 (30.5, 38.3)	0.278
Chloride (mEq/L), Median (Q1,Q3)	110 (106, 114)	110 (106, 114)	109 (104, 114)	<0.001
BUN (mg/dL), Median (Q1,Q3)	45 (28, 72)	41 (26, 65)	56.5 (36, 83)	<0.001
Albumin (g/dL), Median (Q1,Q3)	2.9 (2.6, 3.2)	2.9 (2.68, 3.2)	2.8 (2.4, 3.2)	<0.001
Glucose (mg/dL), Median (Q1, Q3)	191 (148, 266)	186 (144.5, 258.5)	204.5 (158, 282.75)	<0.001
Scr (mg/dL), Median (Q1,Q3)	1.9 (1.2, 3.2)	1.6 (1.1, 2.7)	2.6 (1.6, 3.9)	<0.001
TBIL (mg/dL), Median (Q1,Q3)	1.03 (0.53, 2.3)	1 (0.5, 2)	1.3 (0.6, 3.2)	<0.001
PT (S), Median (Q1,Q3)	18.4 (15, 28.7)	17.2 (14.5, 25.5)	21.45 (16.5, 32.3)	<0.001
Monocytes (%), Median (Q1,Q3)	6.72 (4.6, 9.1)	7 (4.7, 9.31)	6.1 (4, 8.8)	<0.001
Bicarbonate (mEq/L), Median (Q1,Q3)	28 (25, 32)	29 (26, 32)	25 (20, 29)	<0.001
PTT (S), Median (Q1,Q3)	45.2 (34, 92.2)	41.7 (33.1, 85.52)	55.25 (37.5, 106.72)	<0.001
Basophils (%), Median (Q1,Q3)	0.3 (0.1, 0.5)	0.3 (0.2, 0.5)	0.2 (0, 0.4)	<0.001
MCV (%), Median (Q1,Q3)	96 (91, 101)	95 (91, 100)	97 (92, 102)	<0.001
AG (mEq/L), Median (Q1,Q3)	19 (16, 23)	18 (16, 21)	21 (18, 26)	<0.001
pH value, Median (Q1, Q3)	7.37 (7.3, 7.42)	7.37 (7.31, 7.42)	7.35 (7.27, 7.42)	<0.001
SOFA, Median (Q1, Q3)	8 (5, 11)	7 (5, 10)	11 (8, 13)	<0.001

BMI: body mass index; SBP: systolic blood pressure; GCS: glasgow coma scale; AMI: acute myocardial infarction; AKI: acute kidney injury; RBC: red blood cell; WBC: white blood cell; RDW: red blood cell distribution width; ALT: alanine aminotransferase; ALP: alkaline phosphatase; INR: international normalized ratio; HCT: hematocrit; BUN: blood urea nitrogen; Scr: Serum creatinine; TBIL: total bilirubin; PT: prothrombin time; PTT: partial thromboplastin time; MCV: mean corpuscular volume; AG: anion gap.

### Model performance and interpretation

**FIGURE 2:**
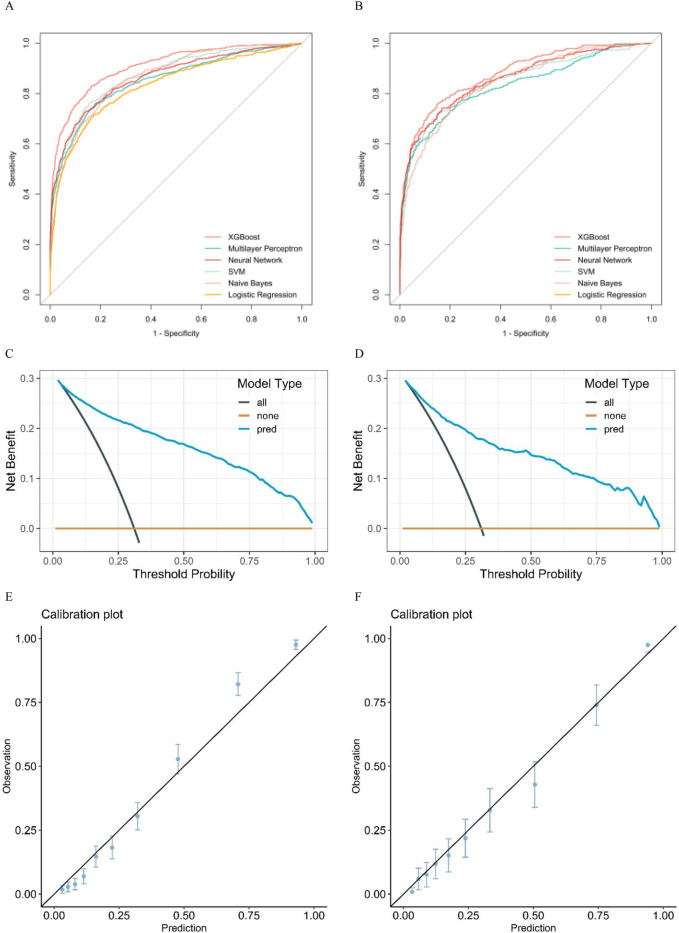
**(A)** pertains to the training set, while **(B)** pertains to the validation set. **(A, B)** display the ROC curves of eight machine learning models used to predict the risk of death in elderly patients with sepsis. Additionally, **(C, D)** show the DCA curves of the XGBboost machine learning model used to predict the risk of death in elderly sepsis patients. **(C)** represents the training set, while **(D)** represents the validation set. Figures **(E, F)** display the calibration curves of the XGBboost machine learning model that was used to predict the risk of death in elderly sepsis patients. Figure E pertains to the training set, whereas Figure F pertains to the validation set.

**FIGURE 3:**
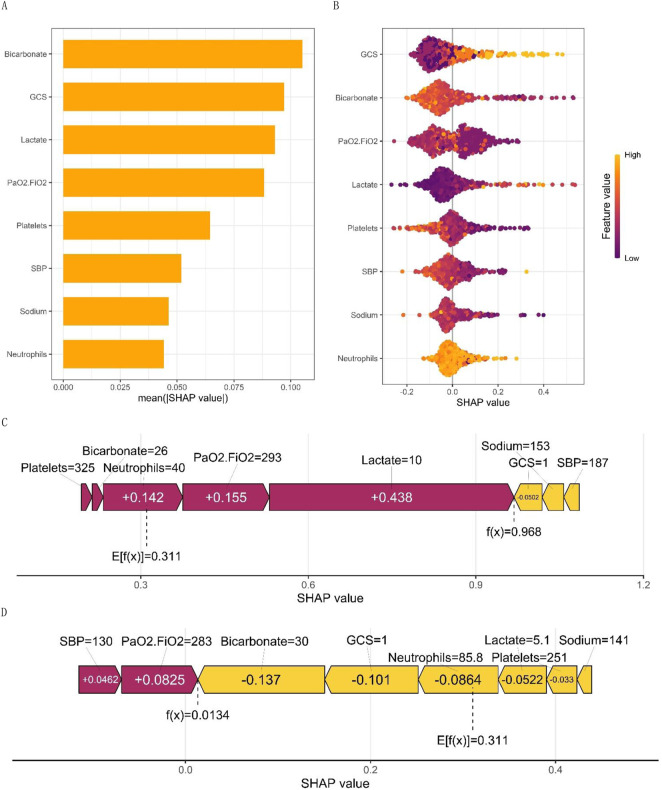
**(A)** displays the SHAP values for macro feature importance, while **(B)** presents a scatterplot of macro feature density. Each row of the plot represents a feature, with the SHAP value as the horizontal coordinate. Features are ranked according to the average absolute value of SHAP, the most important feature of the model. Wide areas indicate a large number of samples clustered together. The diagram employs a dot to represent a sample, with yellow indicating a higher value of the feature and purple indicating a lower value. This generates a ranking graph of the feature’s significance. **(C, D)** show the micro single-sample feature influence diagram.

Six distinctive machine-learning models were constructed on the training dataset, such as logistic regression, neural network, support vector machine, multilayer perceptron, Naive Bayes, and extreme gradient boosting. Table 2, Table 3, and Figures 2A, B present the key metrics of these models on the validation dataset, including sensitivity, specificity, positive predictive value, negative predictive value, recall, F1 score, AUC value, and precision. Based on a comprehensive trade-off comparison, the extreme gradient boosting model demonstrated the optimal prediction performance, achieving an AUC value of 0.88 (0.86.0.90) and a high accuracy rate of 0.84 (0.81.0.86). To investigate the performance and calibration capability of the Extreme Gradient Boosting machine learning model, the DCA curve and calibration curve were plotted for the training and test sets, respectively. In clinical contexts, the term ‘net benefit’ refers to the probability of a patient’s disease occurring being minimized when further medical intervention is deemed necessary (Lee et al., 2021). Figures 2C, 3D display three lines: orange, black, and blue. The orange line represents the net benefit of no treatment for all individuals, which is naturally zero. The black line represents the net benefit of treating all individuals, which decreases as the threshold probability increases. The graph displays the change in net benefit of our decision model at different threshold probabilities. If the blue line closely follows the orange and black reference lines, it may suggest that the model has limited practical application value. However, if the blue line consistently exceeds the reference lines across a wide range of threshold intervals, it may demonstrate that the model has a higher net benefit. The ML prediction model constructed demonstrated good net gains in both the training set and the validation set, as shown in Figures 2C, D. The calibration curve illustrates the model’s predicted probability compared to the actual observation probability in the data (refer to Figures 2E, F). The results indicate that the machine learning model developed in this study exhibits exceptional calibration ability.

**TABLE 2:** Predictive power of eight machine learning models.

CLS	Sensitivity	Specificity	Pos pred value	Neg pred value
XGBoost	0.64 (0.59.0.69)	0.93 (0.91.0.94)	0.80 (0.75.0.84)	0.85 (0.83.0.87)
Neural Network	0.62 (0.57.0.67)	0.93 (0.91.0.94)	0.79 (0.74.0.84)	0.84 (0.82.0.87)
SVM	0.62 (0.57.0.67)	0.91 (0.89.0.93)	0.76 (0.71.0.81)	0.84 (0.82.0.87)
Naive Bayes	0.80 (0.76.0.84)	0.71 (0.68.0.74)	0.55 (0.51.0.60)	0.89 (0.86.0.91)
Multilayer Perceptron	0.61 (0.56.0.66)	0.92 (0.89.0.93)	0.76 (0.71.0.81)	0.84 (0.81.0.86)
Logistic Regression	0.59 (0.54.0.64)	0.92 (0.89.0.93)	0.76 (0.71.0.81)	0.83 (0.81.0.86)

**TABLE 3:** Predictive power of eight machine learning models.

CLS	F1 score	Recall	AUC(95%CI)	Accuracy
XGBoost	0.71	0.64	0.88 (0.86.0.90)	0.84 (0.81.0.86)
Neural Network	0.70	0.62	0.86 (0.83.0.88)	0.83 (0.81.0.85)
SVM	0.69	0.62	0.85 (0.82.0.87)	0.82 (0.80.0.84)
Naive Bayes	0.65	0.80	0.84 (0.82.0.87)	0.74 (0.71.0.76)
Multilayer Perceptron	0.68	0.61	0.83 (0.80.0.86)	0.82 (0.80.0.84)
Logistic Regression	0.66	0.59	0.82 (0.79.0.84)	0.82 (0.79.0.84)

CLS, classifiers; AUC, area under the curve; CI, confidence interval; SVM, support vector machine; XGBoost: Extreme Gradient Boosting.

Figure 3A illustrates the global feature importance mapping generated by inputting the SHAP value matrix into the bar graph function. The plot represents the average absolute value of each feature’s global importance across all samples, while the feature’s criticality in the model is visualized on the Y-axis. Figure 3B presents each patient as a point, with their X-axis coordinates corresponding to the predicted value given by the prediction model. The colors of the dots are changed to represent the values that are predicted by the model. For example, it is worth noting that the Glasgow Coma Scale (GCS) has a significant impact on predicting outcomes. As the GCS value increases, so does the mortality rate. This effect is determined by the level of the feature and is visualized by the yellow and purple dots on the graph. Yellow dots represent high GCS values on the positive side of the X-axis, while purple dots represent low GCS values on the negative side of the X-axis. The model indicated a negative correlation with Platelets, PaO2/FiO2, SBP, and Sodium. Notably, PaO_2_/FiO_2_ exhibited a bimodal distribution, reflecting the differential contributions of distinct patient subgroups, whereas other variables, such as lactate and platelet count, displayed unimodal distributions. This visualization elucidates the relative importance of each biomarker in predicting mortality risk, highlighting potential threshold effects (e.g., the PaO_2_/FiO_2_ threshold for delineating the severity of hypoxemia) and underscoring the complexity of oxygenation indices in sepsis prognosis. Such analysis enhances the interpretability of machine learning models and informs the development of precision medicine strategies tailored to the specific physiological characteristics of individual patients.

Figures 3C, D display the personalized trait attribution analysis for two randomly selected patients. The mean effect value, Ef(x), across all sample data is 0.311. Each characteristic variable’s contribution is visualized as an arrow, with its direction indicating its effect on the probability of the outcome - either decreasing the likelihood of a negative outcome or increasing the chance of a positive outcome. To distinguish between positive (purple) and negative (yellow) effects, they are color-coded. The diagram presents the arrows in an order based on their magnitude of influence on the results. The length of each arrow accurately reflects the strength of influence of each feature, proportional to the SHAP value of the corresponding feature. Figure 3C presents the characteristic attributions of a patient who unfortunately passed away. In contrast, Figure 3D shows the characteristic attributions of a patient who successfully recovered. The identified high-risk factors that increased the risk of mortality were high lactate levels, low PaO2/FiO2, high centrocytes, low platelet count, and low bicarbonate. Although a low PaO2/FiO2 ratio is still considered a risk factor for mortality, it is important to note that this patient’s high bicarbonate levels, low GCS, and high platelet count were found to significantly reduce the risk of death. This comparative analysis highlights the complex interplay of various factors in risk assessment.

**FIGURE 4:**
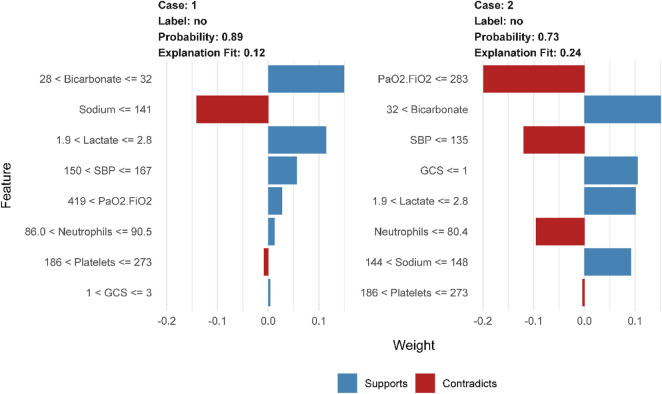
LIME interpretability-based prediction of mortality risk in patients with sepsis: analysis of key physiological indicator weights, supportive/contradictory effects, and clinical decision value in Case1 and Case2.

To further elaborate on our constructed models, we have also employed lime, an interpretable tool commonly used in the field of machine learning (Peng and Menzies, 2021). Figure 4 demonstrates how physicians can improve their decision-making process with the assistance of the model, provided that clear and comprehensible explanations are given. The blue color in the figure represents features that contribute to the predicted outcome, while the red color indicates features that detract from it. The graph illustrates the weight assigned to supporting (blue) or not supporting (red) on the horizontal axis, while the importance of features is ranked on the vertical axis. In the case of the patient who survived, it can be concluded that bicarbonate in the range of 28–32 and lactate in the range of 1.9–2.8 are supportive, as well as a blood pressure between 150–167. The prediction accuracy for this case is high at 0.89, which demonstrates the excellent performance of our prediction model.

**FIGURE 5:**
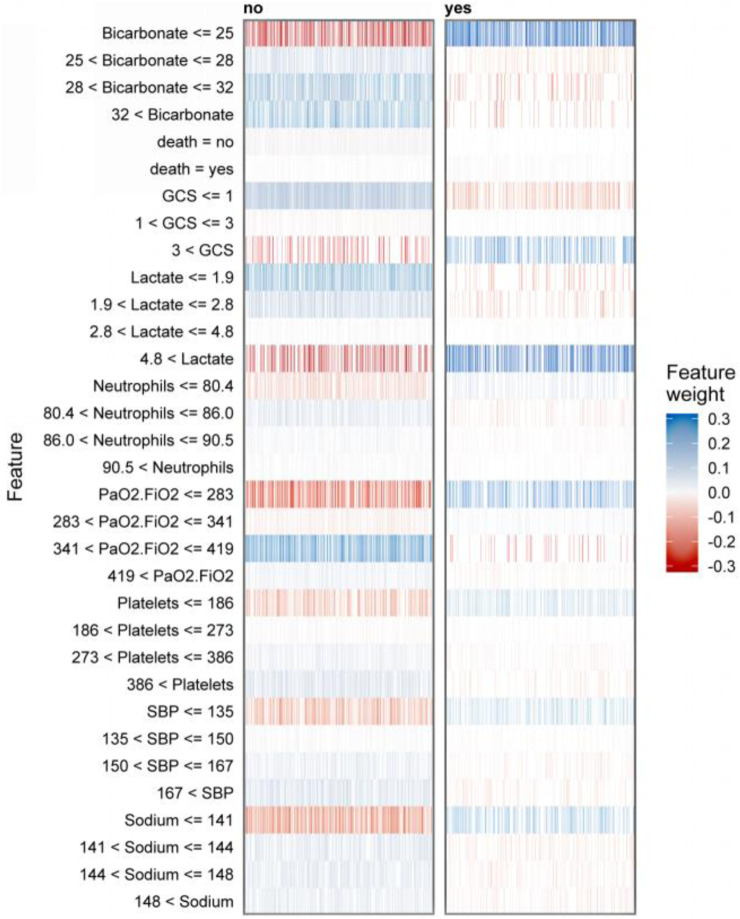
LIME-based interpretable predictive analysis of sepsis death: visualization of dual-category (survival/death) key physiological indicators with multi-interval weight distribution and positive and negative contribution.

Figure 5 presents a global analysis of 2,500 randomly selected patients, demonstrating the range of features and their corresponding weight assignments for these cases. The gradient from red to blue in the figure shows the dynamic evolution of the feature weights. The dark blue region indicates strong support for the predicted outcome, while the increasingly reddish regions suggest a gradual weakening of the support for the predicted outcome. The construction of the prediction model involves feature ranges represented by vertical coordinates. The shade of their color directly maps the importance and influence of the feature in the model. These feature range values may be more suitable for clinicians to make decisions and may be more easily understood.

## Discussion

In this study, a machine learning model was developed and validated to predict short-term mortality risk in elderly sepsis patients. The model is designed to be highly interpretable and easy to understand. This study analyzed 49 variables of elderly sepsis patients in detail after hospital admission, including demographic data, vital signs, and laboratory test indices. A feature recursive elimination algorithm was used to accurately select the eight key feature variables with the highest association with the risk of death from a large number of feature variables for use in constructing the model. The study suggests that the XGBoost algorithm may be more effective than other machine learning algorithms in predicting short-term death risk in elderly sepsis patients. XGBoost is known for its efficiency, flexibility, and widespread use in data mining, medicine, and other fields (Li J. et al., 2022; Hou et al., 2020).

In recent years, machine learning-based predictive models have become increasingly prevalent. However, it is important to acknowledge that these models can be difficult to interpret, often resembling opaque ‘black boxes’ that hinder understanding of the decision-making process, even for those who comprehend the underlying mathematical algorithms (Fleuren et al., 2020; Giannini et al., 2019). We aim to establish trust and encourage the use of our machine-learning predictive models among physicians. To achieve this, we utilized two advanced interpretable analysis techniques, SHAP and lime, to provide a detailed analysis of our XGBoost machine learning predictive model. Our analysis systematically explored the associations between characteristic variables and the risk of death from sepsis in the elderly. We are confident that our approach will provide physicians with the necessary information to make informed decisions (Azodi et al., 2020). The lime interpretable analysis technique has the advantage of representing the weighting of the predictive model accounted for by the ranges of the characteristic variables. This approach is clinically applicable and provides physicians with clear and intuitive decision support.

Figure 5 displays the interpreter that was constructed after screening 2,500 samples. This demonstrates the full range of feature values and their corresponding feature importance weights in the prediction. The figure displays negative values in red and positive values in blue, with the shade of blue indicating the strength of support for the conclusion. It is worth noting that the right side of the graph represents cases where deaths occurred, while the left side represents cases where no deaths occurred. According to the graph, it can be observed that when bicarbonate levels are 25 mEq/L or lower, there is a significant increase in the risk of death for patients. This finding highlights the importance of monitoring bicarbonate levels in patient care. Acid-base imbalances are a common occurrence in critically ill patients, and it is important to address them promptly and effectively (Achanti and Szerlip, 2023). According to a cohort study, there is an association between low bicarbonate levels and increased mortality (Mitra et al., 2020). According to the study, patients with a GCS score greater than three were found to have an increased risk of death. It has been previously suggested by research that hypotension accompanying an abnormal GCS can be a crucial indicator for identifying patients at high risk of sepsis infection (Lane et al., 2020). The study suggests that a lactate value exceeding 4.8 mmol/L is a significant high-risk factor for sepsis patients facing mortality risk. Furthermore, Liu and Yang et al.'s study provides evidence of a strong association between plasma lactate levels and poor prognosis and mortality prediction in sepsis patients (Liu et al., 2019; Yang et al., 2022). In elderly patients with sepsis, a PaO2/FiO2 ratio below 283 is a significant risk indicator for life-threatening conditions. It is crucial to intervene rapidly when the ratio decreases to prevent patient mortality, as confirmed by previous studies (Annane et al., 2017). Platelet count is a critical factor in thrombosis, and thrombocytopenia is a prevalent condition in sepsis patients. Therefore, platelet count serves as a crucial indicator for assessing sepsis severity (Iba and Levy, 2018; Lyons et al., 2018).

There are, of course, limitations to this study. The data used in this study were obtained from publicly available databases, which may not include all the necessary variables. Moreover, the study sample is predominantly from Western countries, which may restrict the applicability of our model to other ethnic groups. Additionally, the retrospective and observational nature of this study may be susceptible to selection bias. We are confident that the model developed in this study can accurately predict the short-term risk of mortality in elderly patients with sepsis.

## Conclusion

We have developed an XGBoost model that is easy to understand and accurately predicts the risk of death in elderly sepsis patients. Our interpretable machine learning tools have helped to identify the risk factors for elderly patients with sepsis, which has increased the confidence of healthcare providers in the predictions. The model’s variable influence ranges and weight assignments are easily understandable, which makes it more practical for clinical applications. This provides physicians with a clear and intuitive basis for decision support. It is worth noting that this feature enhances the model’s credibility and reliability.

## Data Availability

Publicly available datasets were analyzed in this study. This data can be found here: https://physionet.org/content/mimiciv/2.2/. Further inquiries can be directed to the corresponding author.
